# Interaction between SARS-CoV PBM and Cellular PDZ Domains Leading to Virus Virulence

**DOI:** 10.3390/v16081214

**Published:** 2024-07-29

**Authors:** Jose M. Honrubia, Jose R. Valverde, Diego Muñoz-Santos, Jorge Ripoll-Gómez, Nuria de la Blanca, Jorge Izquierdo, Marta Villarejo-Torres, Ana Marchena-Pasero, María Rueda-Huélamo, Ivan Nombela, Mercedes Ruiz-Yuste, Sonia Zuñiga, Isabel Sola, Luis Enjuanes

**Affiliations:** 1 Department of Molecular and Cell Biology, Centro Nacional de Biotecnología (CNB-CSIC), Darwin 3, Campus Universidad Autónoma de Madrid, 28049 Madrid, Spain; 2 Scientific Computing Service, Centro Nacional de Biotecnología (CNB-CSIC), Darwin 3, Campus Universidad Autónoma de Madrid, 28049 Madrid, Spain

**Keywords:** coronavirus, virus–host interaction, virulence, PBM-PDZ

## Abstract

The interaction between SARS-CoV PDZ-binding motifs (PBMs) and cellular PDZs is responsible for virus virulence. The PBM sequence present in the 3a and envelope (E) proteins of SARS-CoV can potentially bind to over 400 cellular proteins containing PDZ domains. The role of SARS-CoV 3a and E proteins was studied. SARS-CoVs, in which 3a-PBM and E-PMB have been deleted (3a-PBM-/E-PBM-), reduced their titer around one logarithmic unit but still were viable. In addition, the absence of the E-PBM and the replacement of 3a-PBM with that of E did not allow the rescue of SARS-CoV. E protein PBM was necessary for virulence, activating p38-MAPK through the interaction with Syntenin-1 PDZ domain. However, the presence or absence of the homologous motif in the 3a protein, which does not bind to Syntenin-1, did not affect virus pathogenicity. Mutagenesis analysis and in silico modeling were performed to study the extension of the PBM of the SARS-CoV E protein. Alanine and glycine scanning was performed revealing a pair of amino acids necessary for optimum virus replication. The binding of E protein with the PDZ2 domain of the Syntenin-1 homodimer induced conformational changes in both PDZ domains 1 and 2 of the dimer.

## 1. Introduction

Coronaviruses (CoVs) recently became a major public health concern due to their pathogenic capacity, ranging from the common cold to more severe diseases such as Severe and Acute Respiratory Syndrome (SARS), Middle East Respiratory Syndrome (MERS), and COVID-19 [1]. SARS-CoV is a member of the *Coronaviridae* family, which initially caused a significant outbreak in 2002–2003, resulting in over 8000 cases and 774 deaths worldwide [2] and, more recently, SARS-CoV-2 caused the COVID pandemic with more than 773 million infected people (https://www.who.int/emergencies/diseases/novel-coronavirus-2019/situation-reports, accessed on 10 June 2024). SARS-CoV originated in bats and was transmitted to humans through civet cats as an intermediate host [3]. SARS-CoV primarily spreads through respiratory droplets and can cause severe respiratory illness, with symptoms including fever, cough, and shortness of breath. Since the initial outbreak, there have been significant efforts to develop treatments and vaccines, particularly after the SARS-CoV-2 outbreak in 2019, to prevent future pandemics.

An important area of research has been the study of specific SARS-CoV protein interactions with host cell proteins including PDZ domains, of 80–90 amino acids found in the signaling proteins of bacteria, yeast, plants, viruses, and animals. PDZ is an acronym combining the first letters of the first three proteins discovered to contain the PDZ domain, the postsynaptic density protein (PSD95), Drosophila disc large tumor suppressor (Dlg1), and zonula occludens-1 protein (Zo-1) [4]. PDZ-binding motifs (PBM) are found in many viral and cellular proteins that can interact with cellular PDZ domains, which are in turn found in many intracellular signaling proteins. These interactions are important for regulating a wide range of cellular processes, including cell proliferation, differentiation, and apoptosis [5]. In particular, SARS-CoV 3a and E proteins have a PBM formed by the four C-terminal residues, 3a PBM (SVPL), E PBM (DLLV), that can potentially bind over 400 cellular proteins containing a PDZ domain, giving them a significant role in the control of cell function [5].

A comparative study of the functional motifs included within the SARS-CoV 3a and E proteins showed that the two full-length proteins were required for maximum SARS-CoV replication and virulence [6]. A virus missing both of them was not viable, whereas the presence of at least one of these two proteins with a functional PBM restored virus viability. E protein PBM was essential for virulence due to the activation of the p38-MAPK route through its interaction with the PDZ domain of Syntenin-1 [7], whereas the presence or absence of the homologous motif in 3a protein, unable to bind to Syntenin-1, did not influence virus pathogenicity [6].

The functional relevance of SARS-CoV proteins 3a and E in virus replication and virulence, mediated by their interaction with PDZ domain-containing proteins, is studied using a combination of in vitro, in vivo, and in silico techniques to examine the effect of the binding of their carboxyterminal PBM to PDZ-containing proteins. This study provides new insights into the mechanisms of SARS-CoV pathogenicity and may have implications for the development of antiviral therapies. E protein of SARS-CoV plays a crucial role in virus virulence. Its absence fully attenuates the virus [8,9]. In addition, residues 20 and 22, counting from the carboxy-terminus of this protein influenced optimum virus replication, as mutation of each of them significantly reduced SARS-CoV replication. In addition, in silico results predicted that the binding of the PBM of SARS-CoV E protein to the PDZ2 domain of Syntenin-1 was sufficient to induce conformational changes in the PDZ1 domain, highlighting the relevance of this interaction in regulating the function of Syntenin-1 and its effect in virulence.

## 2. Materials and Methods

### 2.1. Ethics Statement

The animal experimental protocols were approved by the NIH-CDC, the Environmental Council of Madrid (permit number: PROEX 146.6/20), and the Ethical Committee of the Center for Animal Health Research (CISA-CSIC) (permit numbers: CBS 2014/005 and CEEA 2014/004), in accordance with Spanish National Royal Decree (RD 53/2013), international EU guidelines 2010/63/UE, and Spanish National law 32/2007 on the protection of animals used for experimentation and other scientific purposes. The infected animals were housed in a self-contained ventilated rack (Allentown, NJ, USA) in a biosafety level 3+ (BSL3+) laboratory of the Center for Animal Health Research (CISA-CSIC, Madrid, Spain).

### 2.2. Cells

Vero E6 cells (ATCC, CRL-1586) were grown at 37 °C with an atmosphere of 98% humidity, in Dulbecco’s modified Eagle medium (DMEM) supplemented with 25 mM HEPES, 2 mM L-glutamine, 1% non-essential amino acids and 10% fetal bovine serum (FBS).

### 2.3. Generation of Recombinant Virus Infectious Clones

The SARS-CoV-MA15 cDNA was assembled in a pBAC (pBAC-SARS-CoV-MA15) [10] and used to introduce the corresponding mutations affecting E protein. To generate viruses with mutations in SARS-CoV E protein carboxy-terminus, DNA fragments including nucleotides 26,017 to 26,884 of SARS-CoV-MA15 genome (GenBank accession FJ882957) flanked by BamHI and XcmI restriction sites were generated by overlapping PCR using oligonucleotides SARS-E-VS (5′-CTCTTCAGGAGTTGCTAATCCAGCAATGG-3′) and SARS-26885-RS (5′-GGTCCTTAATGTCACAGCGCCC-3′) and the mutated oligos indicated in Table 1.

These fragments carried different mutations located on E gene generating amino acid changes at E protein carboxy-terminus: E-A[27,29], E-A[20,22], E-A[17,18], E-A[13,14], E-A[10,11], E-A[8,9], E-G[1], E-G[2], E-G[3], E-G[4], E-G[5,6], E-G[8,9,10]). The fragments were digested with enzymes BamHI and XcmI and introduced into an intermediate plasmid pBAC-BamHI-RsrII-SARS-CoV containing nucleotides 26,044 to 29,783 of the SARS-CoV genome. Next, these plasmids were digested with enzymes BamHI and RsrII, and the fragments containing the different mutations were inserted into plasmid pBAC-SARS-CoV-MA15 digested with the same enzymes, obtaining the respective infectious clones. The integrity of cDNAs was verified by restriction analysis pattern and by Sanger sequencing.

### 2.4. Recovery of Recombinant Virus Variants from cDNA Clones

Vero E6 cells were grown to 95% confluence in 12.5 cm^2^ flasks and transfected with 6 µg of each infectious SARS-CoV cDNA clone, and 18 µL of Lipofectamine 2000 (Invitrogen, Waltham, MA, USA). At 6 h post-transfection (hpt), the transfection medium was replaced by standard Dulbecco’s medium with 10% FBS, and incubated at 37 °C for 72 h. Cell supernatants were harvested and passaged once on fresh cells, and the recovered viruses were cloned by three rounds of plaque purification, following standard procedures for SARS-CoV [11]. The supernatants were collected at 72 hpi and titrated. The 3′ end genome of the different viruses, including all structural and accessory genes and the 3′UTR, were sequenced. All sequences were compared to that of the parental wild-type (WT) virus sequence using SeqMan 17.4 software (Lasergene, Madison, WI, USA).

### 2.5. Growth Kinetics

Sub-confluent monolayers (around 90% confluency) of Vero E6 were infected at a MOI of 0.001 with parental viruses or the respective mutant viruses. Culture supernatants were collected at 24, 48, and 72 hpi, and virus titers were determined as previously described [11].

### 2.6. Mice

To determine mutant virus pathogenesis, SARS-CoV mutant viruses were inoculated into 16-week-old BALB/c OlaHsd (Harlan, West Lafayette, IN, USA). Mice were anesthetized with isoflurane and inoculated intranasally using 100,000 pfu of the corresponding virus in a 50 μL volume of DMEM supplemented with 2% FBS. Manipulation of infected mice was carried out in a level 3+ biological containment laboratory at CISA-CSIC (Madrid, Spain) equipped with the required containment infrastructure for animal and cell cultures work.

### 2.7. Lung Viral Titers of Infected Mice

Lungs from infected mice were harvested on different days post-infection (dpi). Lungs were homogenized in 2 mL of PBS containing 100 IU/mL penicillin, 0.1 mg/mL streptomycin, 50 µg/mL gentamicin, and 0.5 µg/mL amphotericin B (Fungizone), using gentleMACS Dissociator (Miltenyibiotec, Bergisch Gladbach, Germany). Virus titers were determined as previously described [11].

### 2.8. RNA Analysis

Total RNA was isolated from cell culture supernatant or lungs using the RNe-asy mini kit (Qiagen, Hilden, Germany) following the manufacturer’s instructions. cDNA was generated using the High Capacity cDNA RT kit reagent (Applied Biosystems, Waltham, MA, USA), following the manufacturer’s recommendations. To verify the sequence of the mutations introduced into the viral genome, 4 μL of the cDNA generated in the RT reaction was used as a template in a PCR reaction using the enzyme Taq Polymerase (Invitrogen).

### 2.9. Binding of SARS-CoV E Protein to Syntenin-1

We obtained from UniProt the 15 amino acid carboxyterminal sequences of human SARS-CoV and of syndecan (to be used as a reference baseline) [12]. We used Galaxy PepDock to derive an ab initio structure for each of these peptides and dock it to a Syntenin-1 tandem [13]. The predicted conformers were inspected to select the most biologically significant ones, and these were finally subjected to additional refinement consisting of a GROMACS energy minimization in vacuum using the AMBER force field [14], followed by an energy minimization in 150mM solution using the OPLS-AA force field [15]. To obtain a more accurate affinity estimation, we calculated clashes, contacts and H-bonds, total system energy, repulsion energy, and average of the scores from X-score [16], DSX [17], Vina [18], DL-SCORE [19], and NN-SCORE [20].

### 2.10. Statistical Analysis

Two-tailed, unpaired Student’s *t* tests were used to analyze the differences in mean values between groups. The statistical significances were indicated as follows: * *p* < 0.05; ** *p* < 0.01; *** *p* < 0.001.

## 3. Results

### 3.1. Role of the PBM Sequence of 3a and E Proteins in the Viability and Virulence of SARS-CoV

**Figure 1:**
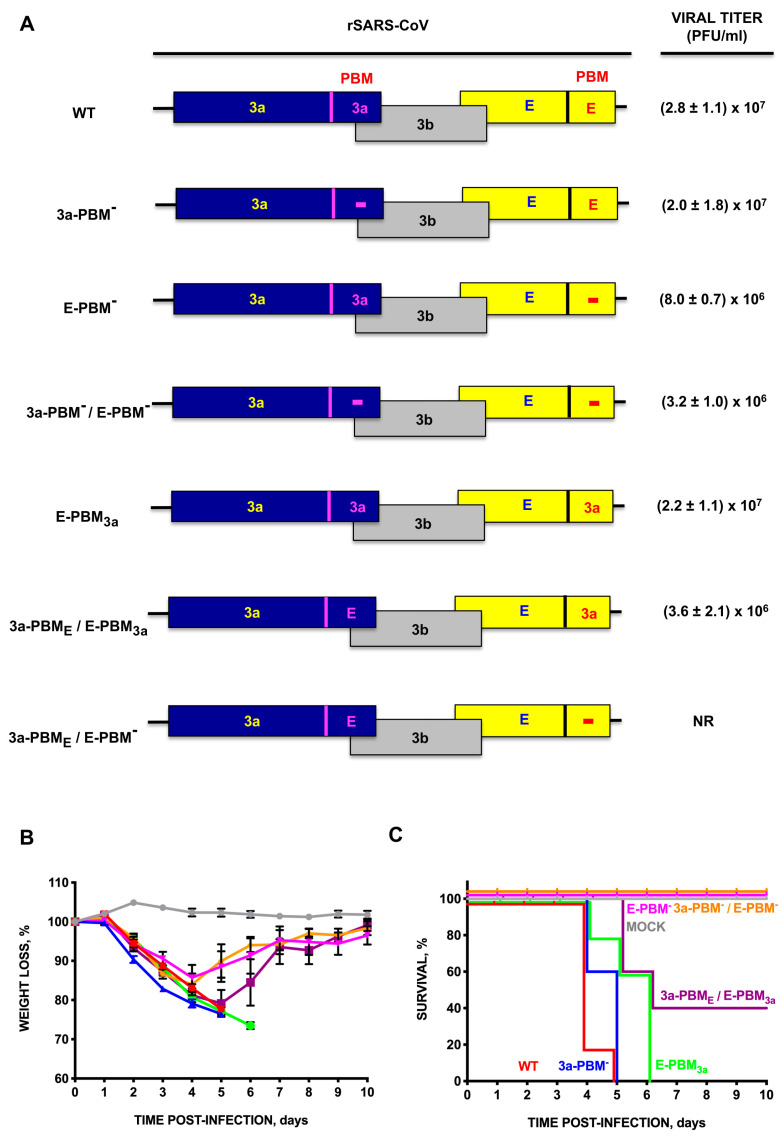
Virulence of SARS-CoV mutants in which the 3a and E protein PBMs have been deleted or exchanged. (**A**) Scheme of mutants generated by combining mutations in the PBM of 3a (SVPL) and E (DLLV) proteins. Optimum viral titers are shown on the right. Mean weight loss (**B**) and survival (**C**) of 16-week-old infected Balb/c mice were monitored for 10 days and compared with those of mock-infected mice. Error bars represent the standard deviation of mouse weight for each experimental variable. Data shown are the mean and standard deviation obtained in three experiments. Uninfected mice, gray circles. Mice inoculated with 100,000 pfu of parental virus (WT, red), or with mutant viruses generated by reverse genetics, without the PBM of 3a protein (3a-PBM-, blue), without the PBM of E protein (E-PBM-, pink), without both motifs (3a-PBM-/E-PBM-, orange), with the PBM of 3a protein in the context of E protein (E-PBM3a, green) or with the PBM of both proteins swapped (3a-PBME/E-PBM3a, purple).

To analyze the role of the PBM sequence of 3a and E proteins in SARS-CoV viability and virulence, a collection of mutants was assembled combining some mutants previously reported [6], with others specifically generated for this manuscript in which PBMs from 3a and E proteins were present or deleted. In the first two mutants generated in the PBM, 3a-PBM- and E-PBM-, each PBM sequence was deleted, whereas in a second set of deletion mutants, 3a-PBM-/E-PBM-, both PBMs were deleted. In an additional set of deletion mutants, the PBM of E protein was substituted with that of 3a protein (E-PBM3a), or the 3a-PBME and E-PBM3a motifs were interchanged and, in an additional set of mutants, the PBM of 3a protein was replaced by that of E protein, producing the 3a-PBME/E-PBM- mutant (Figure 1A).

All mutants were viable except the combination of 3a-PBME, E-PBM-. Viral titers decreased around 10-fold when the PBMs of both proteins were simultaneously eliminated or swapped. The relevance of the PBM residues of SARS-CoV E protein and adjacent amino acids in the virulence of SARS-CoV was determined in mice intranasally inoculated with the parental virus or with each of the mutants. Weight loss and survival were monitored for 10 days post-infection (dpi). When E protein conserved its natural PBM, independently of the presence or not of 3a-PBM (WT or 3a-PBM-), the virus was highly virulent, similar to when the PBM of E protein was substituted by that of 3a protein (E-PBM3a) (Figure 1B,C). In contrast, mortality in infected mice significantly decreased when the PBM of E protein was deleted (E-PBM-, 3a-PBM-/E-PBM-) or when the PBMs of both proteins were interchanged (3a-PBME/E-PBM3a). These results indicated that there is a hierarchy between the 3a and E proteins and the nature of their PBMs and that the absence of the native PBM of E protein led to the loss of SARS-CoV virulence (Figure 1). The presence of E-PBM in a different protein (3a-PBME/E-PBM3a) significantly reduced de virulence, suggesting that the sequence context of E-PBM is contributing to its biological activity.

### 3.2. Extension of SARS-CoV E Protein PBM

**Figure 2:**
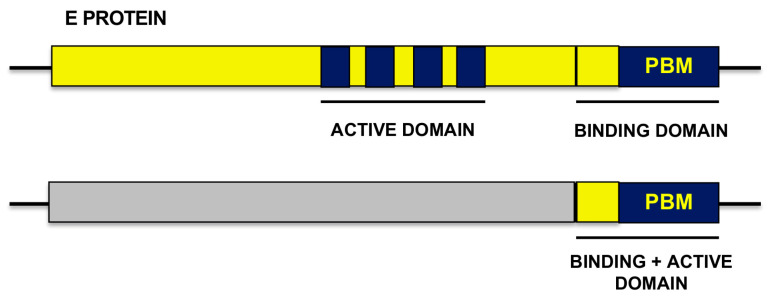
Potential structure of the active domain included within E protein that may be required for the PBM function. Two different structures were considered. One in which, in addition to the PBM core formed by the four carboxyterminal amino acids, E protein contained a hypothetic active domain located to the 5′ side of the PBM (top bar). A second one in which the two domains (PBM and active domain modulating the activity of the first one), overlapped at the carboxy-terminal end (bottom bar).

The relevance of SARS-CoV E protein carboxy-terminal residues and the potential contribution to virulence of the additional N-terminus residues were addressed. Specifically, we asked whether in addition to the four carboxy-terminal residues of E protein, other residues mapping upstream of the PBM also modulate PBM motif activity in the complex PBM-PDZ, and could promote the activation of p38-MAPK leading to virus pathogenesis [7] (Figure 2). Frequently, during the interaction of two proteins, one of its domains is responsible for providing the physical interaction between the two proteins first, and then, the biological consequences are observed. This is the case of the specific cleavage of a DNA or RNA molecule by a DNAse or an RNAse, first, the specific binding of the two molecules takes place, due to the recognition of a specific sequence, and then the specific cleavage by the enzymatic activity cuts the molecules. We considered that a possibility during the binding of the PBM to the PDZ motif also could require two steps, the specific recognition of a structural domain, followed by a specific structural change leading to biological consequences. We considered whether there was an “active domain” located in the N-terminus of the main amino-terminal core of the E protein (Figure 2).

**Figure 3:**
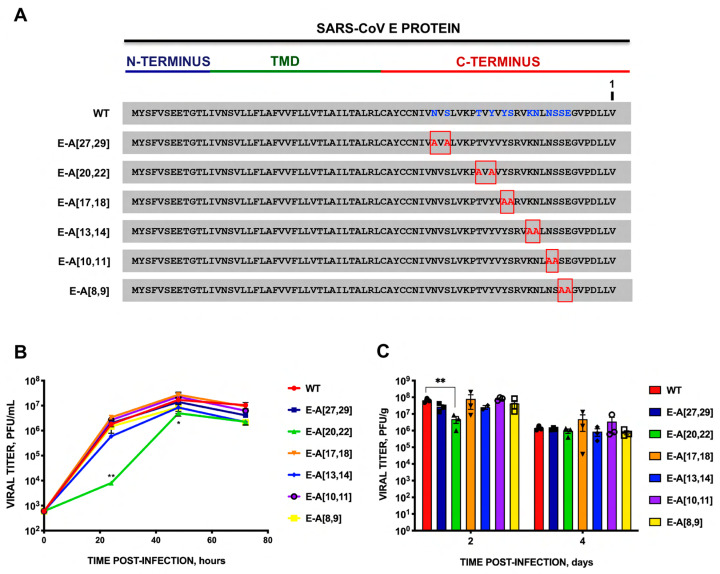
Growth kinetics of SARS-CoV mutants with double mutations of the polar residues of the carboxyterminal end of E protein replaced by alanine. (**A**) Sequence of the parental virus (WT) E protein, and that of the mutant viruses in which two polar residues of the carboxy-terminal end have been mutated to alanine (E-A[X,X], where X is the position of the mutated residue starting from the carboxyl-terminal end of E protein, indicated as 1 in the figure. (**B**) Vero E6 cells were infected at a moi of 0.001 with parental SARS-CoV (WT) (red circles) and with rSARS-CoV-E-A[27,29] (dark blue squares), E-A[20,22] (green triangles), E-A[17,18] (orange inverted triangles), E-A[13,14] (blue diamonds), E-A[10,11] (purple circles) or E-A[8,9] (yellow squares). Supernatants were collected at 24, 48, and 72 h post-infection (hpi) and titrated by the lysis plaque formation method. These results are derived from three independent experiments. (**C**) Groups of six 16-week-old Balb/c mice were intranasally inoculated with 100,000 pfu/mouse of the indicated rSARS-CoV. At 2 and 4 days post-infection (dpi), three mice per group were sacrificed, and viral titers in the lungs were analyzed. Error bars represent the standard error of the mean. * *p*-value < 0.05; ** *p* value < 0.01).

**Figure 4:**
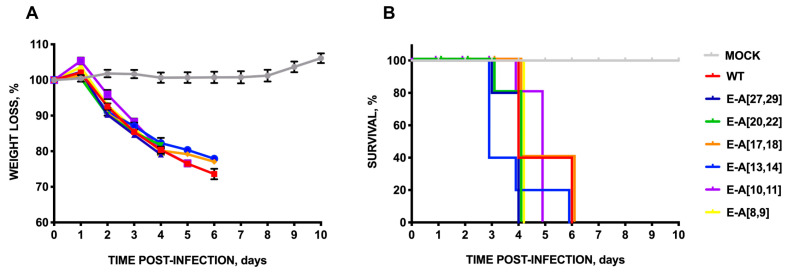
Virulence of SARS-CoV mutants with double substitutions of polar residues of the carboxyterminal E protein by alanine. Groups of five 16-week-old Balb/c mice were intranasally inoculated with DMEM (Mock, gray), or infected with 100.000 pfu/mouse of the parental virus (WT, red), or with the engineered rSARS-CoV: E-A[27,29] (dark blue), E-A[20,22] (green), E-A[17,18] (orange), E-A[13,14] (blue), E-A[10,11] (purple) or E-A[8,9] (yellow). (**A**) Weight loss and (**B**) survival were determined for 10 dpi. Vertical bars represent the standard error of the mean weight of the mice.

To address this question, an alanine scanning was performed on exposed polar amino acid pairs at the carboxyterminal end of E protein: rSARS-CoV-MA15-E-A[27,29], -E-A[20,22], -E-A[17,18], -E-A[13,14], -E-A[10,11], -E-A[8,9] (Figure 3A). The growth kinetics in cell culture (Figure 3B) and mice lungs (Figure 3C) were determined. Only the amino acid pairs located at positions 20 and 22 of E protein, counting from the carboxy-terminus, were necessary for optimum virus replication in cell cultures since mutant E-A[20,22] showed significantly lower titers compared to the parental virus at early time points, whereas the introduction of alternative alanine substitutions in other positions did not cause significant differences in virus replication. This reduction in virus titer was also observed in mice lung at two but not at four days. In contrast, the virulence of the substitution mutant E-A[20,22] was not significantly affected in terms of weight losses (Figure 4A) or survival (Figure 4B), suggesting that these mutations did not cause a strong effect on virus pathogenicity.

### 3.3. Relevance of Alternative E Protein Residues in the PBM or in the Proximal Flanking N-Terminus of the PBM Core Motif, in SARS-CoV Pathogenesis

**Figure 5:**
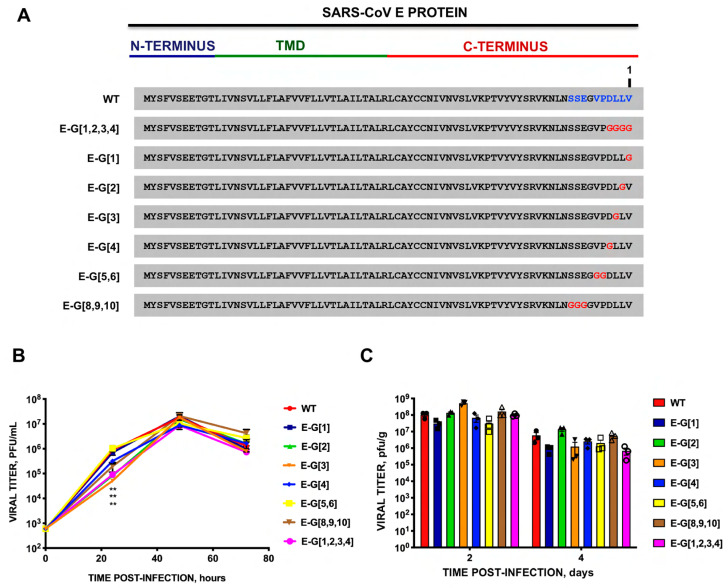
Growth kinetics of SARS-CoV mutants with amino acid substitutions by glycine in carboxyterminal E protein residues that may be relevant in the interaction of the E PBM with cellular Syntenin-1, according to the developed in silico model. (**A**) The E protein sequence of the parental virus (WT) and that of the glycine substitution mutants engineered is shown. (E-G[X], where X is the position of the mutated residue from the carboxy-terminal end of E protein, numbered as in Figure 3A. (**B**) Vero E6 cells were infected at a moi of 0.001 with parental SARS-CoV (WT) (red circles) and with mutants E-G[1] (dark blue square), E-G[2] (green triangle), E-G[3] (orange inverted triangle), E-G[4] (blue diamond), E-A[5,6] (yellow square), E-G[8,9,10] (brown inverted triangle) or with E-G[1,2,3,4] (pink circle). Culture supernatants were collected at 24, 48, and 72 hpi, and titrated by plaque assays. These results are derived from three independent experiments. (**C**) Groups of six 16-week-old Balb/c mice were intranasally inoculated with 100,000 pfu/mouse of rSARS-CoVs. At 2 and 4 dpi, three mice per group were sacrificed, and viral titer in the lung was analyzed. Vertical bars represent the standard error of the mean. **, *p*-value < 0.01.

**Figure 6:**
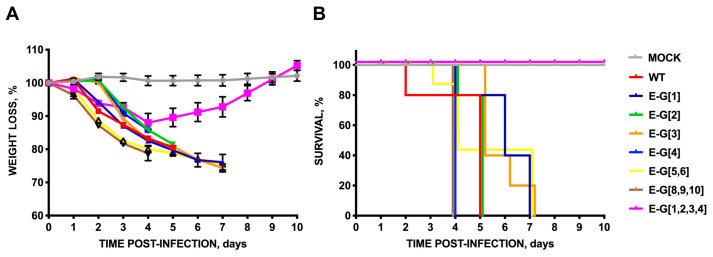
Virulence of SARS-CoV mutants with glycine substitutions in the carboxy-terminal residues of the E protein that may participate in the interaction of this protein with Syntenin-1 according to the structure proposed by the in silico model. Groups of five 16-week-old Balb/c mice were intranasally inoculated with DMEM (Mock, gray), or with 100,000 pfu/mouse of the parental SARS-CoV virus (WT), or with the glycine replacement mutants generated: E-G[1] (dark blue), E-G[2] (green), E-G[3] (orange), E-G[4] (blue), E-G[5,6] (yellow), E-G[8,9,10] (brown), E-G[1,2,3,4] (pink). Weight loss (**A**) and survival (**B**) were monitored for 10 dpi. Vertical bars represent the standard error of the mean weight of the mice.

To further identify the relevance of the carboxy-terminal residues of E protein, potentially involved in the interaction between the virus PBM and the Syntenin-1 PDZ, a collection of mutants was generated in which residues of E protein potentially involved in this binding were replaced by glycine (Figure 5A). This change to G is based on an in silico modeling of the interaction between E protein PBM and Syntenin-1 PDZ. Single mutants of the PBM (rSARS-CoV-MA15-E-G[1], -E-G[2], -E-G[3], -E-G[4]), or double and triple mutants, in which adjacent residues were also substituted, were constructed, because of their proximity to the PBM core. A recombinant virus lacking the PBM of E protein was used as a control by replacing the four amino acids of the PBM with glycine (E-G[1,2,3,4]) (Figure 5A). The results showed that there were limited differences in virus titers (Figure 5B,C). All mice infected with the mutant lacking the E protein PBM (E-G[1,2,3,4]) regained weight and survived. However, all mice infected with the parental virus or with the other mutants lost weight (Figure 6A) and died between 4 and 7 dpi indicating minor differences in the pathogenicity of the different mutants (Figure 6B). These results indicated that the four residues could act as a virulence factor that the PBM of SARS-CoV E protein is a virulence factor. However, individual glycine substitutions within the PBM core, as well as double or triple replacements of adjacent amino acids outside the main core, were not sufficient to generate mutants with an attenuated phenotype, as observed in the case where four PBM core residues were replaced by glycine (Figure 6).

### 3.4. Interaction between the PBM of SARS-CoV E Protein and the PDZ Domain of Syntenin-1

We previously showed that the interaction of the PBM of E protein with the cellular protein Syntenin-1 induces phosphorylation and activation of p38-MAPK, leading to the pathogenesis of SARS-CoV [7]. Human Syntenin-1 has two PDZ domains (PDZ1 and PDZ2) and forms an inverted homodimer in which the PDZ1 domain of one subunit is facing the PDZ2 domain of the other subunit [21].

To determine whether the PBM of SARS-CoV E protein has a preference for either Syntenin-1 PDZ1 or PDZ2, a model of the structure of the interaction between these two proteins was predicted docking the PBM against both PDZ domains using different methods that resulted in over 25,500 poses that were inspected to select the 25 most biologically relevant according to the number of contacts between proteins and affinity of the binding. As docking only allows limited flexibility, we allowed each Syntenin-1 PDZ and E protein PBM to adapt to each other in a realistic environment using a simulation of their molecular dynamics over time thus, obtaining improved conformations prior to analyzing the strength of the interaction. We used several docking methods to obtain various estimations [16,17,18]. The results of the different programs indicated a preference for PDZ2 over PDZ1: Xscore: 8.25 vs. 7.91, DSX: 138.62 vs. 129.13, Vina: 4.93 vs. 4.45 (where more negative values correspond to higher affinity), and greater counts of interaction-stabilizing H-bonds (16 vs. 8) and contacts (257 vs. 84).

**Figure 7:**
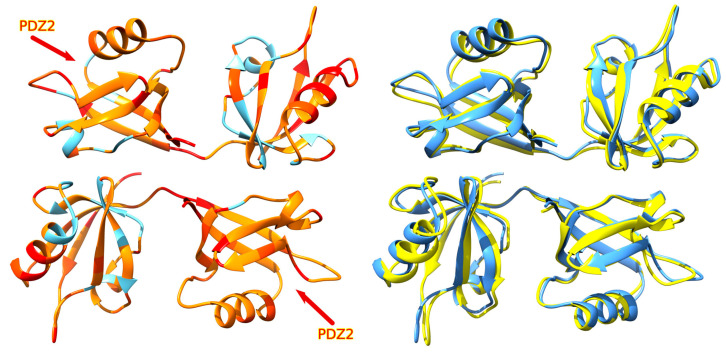
Effect of carboxyl-terminal binding of SARS-CoV E protein on the structure of Syntenin-1. The left panel shows free Syntenin-1 (blue) with regions that change colored by their variance (orange < orange-red < red) calculated by THESEUS, with both PDZ2 domains indicated by arrows. The right panel shows free Syntenin-1 (blue) superposed to the bound form (yellow).

The structural changes induced on Syntenin-1 by E protein PBM binding were studied superposing a free Syntenin-1 homodimer (PDB:1N99) with a model of a Syntenin-1 homodimer bound to the carboxyterminal 10 amino acids of E protein, using the program THESEUS [22], and measuring the distances between the alpha-carbon atoms of key amino acids in each PDZ of every conformation. Binding to PDZ2 in one subunit induced changes in both subunits, with the PDZ2 pocket opening about 2 Å on average, and the unbound PDZ1 pocket opening about 1.3 Å on average (Figure 7). In the best model obtained, the observed distance between the Gly residues in two E decapeptides bound to opposite PDZ2 domains of a Syntenin-1 dimer was 49 Å.

**Figure 8:**
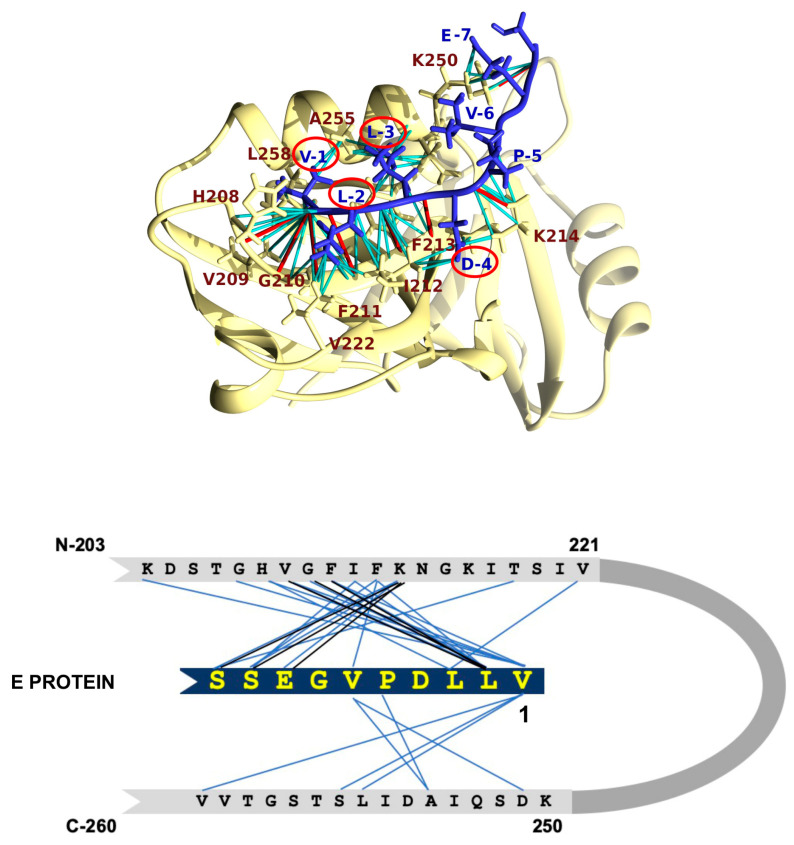
Proposed conformation of the binding between the carboxy-terminus of E protein and Syntenin-1. At the top, the PDZ2 domain of human Syntenin-1 is depicted in yellow, with the amino acids involved in binding to E protein in brown. The carboxyl terminus of SARS-CoV E protein is shown in blue and the four main residues of the PBM are circled in red. Hydrogen bridges are depicted in red and the contacts between the residues of both proteins are in cyan. The same conformation is shown schematically at the bottom. The last 10 residues of the carboxyl-terminal protein are located in the center of the pocket of the PDZ2 domain of Syntenin-1. Direct contacts between the residues are shown in blue and hydrogen bonds in black.

To identify the most relevant residues in the interaction and to determine if residues other than those of the PBM core (E protein terminal four amino acids) might play a complementary role, additional docking and affinity analyses were performed using peptides of 6, 10, and 15 amino acids long, from the carboxy-terminal end of E protein (Table 2). The analysis of the best PBM-PDZ structures selected when considering 10 or 15 aa (Table 2) showed that a major fraction of the interaction energy is contributed by the four core amino acids of the PBM and that increasing peptide length to 10 or 15 amino acids would have a relatively lower impact, although additional residues, in positions −5 to −10 from the carboxy-terminal end, could also contribute to stabilize the complex with H-bonds and contacts (Figure 8). As an example, the Xscore program predicts interaction energies of 9.2, 9.4, and 9.9 kcal/mol for 6, 10, and 15 amino acid peptides, respectively. Increasing negative values indicate a larger release of energy with longer lengths, although the variation is not proportional (Table 2).

Taking into account the distribution of hydrogen bonds and contacts between the two proteins (PBM and Syntenin-1), Leu-2 and Val-1, seem to be the most relevant residues in the PBM. Hydrogen bonds between the PBM and Syntenin-1 are essentially established with Val-1, depending on the relative residue orientations which, according to molecular dynamics simulations, could change over time. A selected representative conformation obtained with the PDZ2 domain of Syntenin-1 and the last 10 amino acids of E protein with the most relevant hydrogen bonds and contacts observed are represented in Figure 8.

## 4. Discussion

### 4.1. Relevance of 3a and E Protein PBM Sequences in SARS-CoV Replication and Virulence

The influence of the PBMs of the SARS-CoV 3a and E proteins lies to a large extent in their potential interaction with cellular proteins with PDZ domains [5,7,23]. These interactions are involved in multiple cellular activities, and their alteration has significant effects on the behavior of viruses of different families [24,25,26,27]. For example, the PBM of the human papillomavirus (HPV-16) E6 oncoprotein interacts with many cellular proteins with PDZ motifs, facilitating tumorigenesis and virus dissemination [28]. Additionally, the F11 protein of the vaccinia virus has both a PDZ domain and a PBM that mediate virus dissemination [29]. Recently, it has been described that the PBM of the West Nile virus (WNV) non-structural protein 5 (NS5) is important for the replication of this virus [30].

The PBM of E protein is involved in SARS-CoV virulence, in contrast to the PBM of 3a protein [5]. Our results confirmed that these motifs have different relevance in pathogenesis, with the PBM of the E protein being associated with replication and virulence to a greater extent than that of the 3a protein. On the other hand, the PBM of the 3a protein becomes critical for virus viability when the E protein is deleted [5]. This difference in relevance between the two PBMs is reinforced by the observation that in the absence of the E protein original PBM, the 3a protein with a functional E protein PBM (E-PBM-/3a-PBME) has a negative and deleterious effect on SARS-CoV viability and replication. In contrast, the PBM of 3a protein is able to reproduce the parental phenotype when it is in the context of E protein (E-PBM3a) (Figure 1). This differential behavior of the deletion mutants missing either the PBM from the 3a or the E protein mutants, could be due to their ability to interact with different cellular proteins including those with a PDZ motif and, also could be due to a different cell compartment localization. In fact, the E protein accumulates in the ERGIC, whereas the 3a is located in membranous structures inside infected cells [6,31,32,33]. These results highlight the complexity of the interactions between viral PBMs and cellular PDZ domains since their role not only depends on the extent of the interactions but also on the target proteins and the cellular compartment in which these interactions take place.

### 4.2. Extension of the PBM of SARS-CoV E Protein

**Figure 9:**
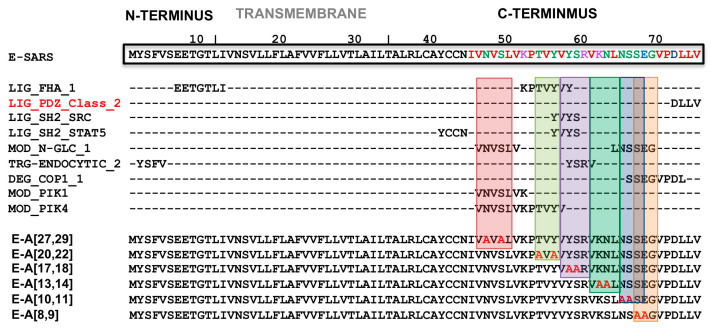
Predicted functional motifs in SARS-CoV E protein affected by alanine substitution in polar residues. The full-length sequence and structural domains of SARS-CoV E protein are shown in the top bar. In the left column, different predicted functional motifs in the E protein of SARS-CoV are indicated [35]. Color coding of amino acids (aa): in red, small hydrophobic aa (including aromatic aa except for tyrosine); in blue: acidic aa; in magenta: basic aa (except histidine); in green, aa with hydroxyl, thiol, or amine groups (glycine is also included). The location of the functional motifs identified in the E protein and the mutants with two polar residues at the carboxyl end substituted by alanine (E-A[X,X], where X is the position of the mutated residue starting at the carboxyl-terminal end of the E protein, are shown. Vertical colored rectangles indicate the location of predicted SARS-CoV-E protein motifs.

PBM-PDZ interactions, in principle, could extend beyond the four amino acids of the PBM, since adjacent amino acids of the PBM also may interact with PDZ domains [34]. In this work, we studied whether the effect of SARS-CoV E protein PBM on virus viability, replication, and virulence was influenced only by the four carboxyterminal residues of the PBM alone, or whether alternative residues at the N-terminus of the main motif could also affect virus viability. To clarify this possibility, recombinants of SARS-CoV with double mutations, affecting various polar residues of the carboxyl terminus that are frequently exposed, were constructed (Figure 3A). All substitution mutants were virulent in the mouse model. Therefore, no functional motif has been identified so far in the carboxyl terminus, apart from the four PBM amino acids, that were relevant to the virulence of SARS-CoV. However, the E-A[20,22] replacing mutant had the lowest viral titer both in vitro and in vivo indicating that these residues are relevant in the infection cycle of SARS-CoV. It was predicted that in the E-A[20,22] mutant, two functional motifs were affected by these mutations: (i) the FHA domain binding motif (FHA-BM) and (ii) phosphotyrosine ligands bound by SH2 domains (Figure 9) [35]. The functional FHA-BM C-terminal carboxyl group is conserved in SARS-CoV and MERS-CoV. FHA domains are the only signaling domains that specifically recognize phosphothreonine (pThr) residues [36,37,38]. This type of protein–phosphoprotein interaction is present in a wide variety of prokaryotic and eukaryotic proteins [39] and is involved in various cellular functions such as signal transduction, vesicular trafficking, and cell cycle control [40,41,42,43,44,45].

The matrix protein (M) of Newcastle disease virus (NDV) is responsible for optimal virus replication through the reduction of TIFA expression levels (a protein with an FHA domain that interacts with TRAF6) [46]. Likewise, it has been described that Herpes Simplex virus type 1 (HSV-1) encodes the viral E3 ubiquitin ligase (ICP0), which promotes transcription, replication, and viral production by binding to the FHA domain of the cellular E3 ubiquitin RNF8 protein [47]. Additionally, previous laboratory results have shown that a rMERS-CoV mutant that also lacked the FHA-BM motif in its E protein had lower titers in the brains of susceptible mice, although not in the lungs, and this was associated with attenuation of MERS-CoV [35]. Furthermore, a deletion mutant of SARS-CoV, in which the amino acids corresponding to the FHA domain region have been eliminated, also showed significantly lower titers [48]. Our findings reinforce the hypothesis that the FHA domain binding motif in human CoV E protein is related to the level of replication of these viruses.

### 4.3. Interaction between the PBM of SARS-CoV E Protein and the PDZ Domain of Human Syntenin-1

The PBM of the SARS-CoV E protein interacts with the PDZ domain of the cellular protein Syntenin-1, inducing the phosphorylation and activation of p38 MAPK, increasing the production of proinflammatory cytokines, and leading to the pathogenesis and virulence of SARS-CoV [7]. To characterize the interaction of E protein and Syntenin-1, we have developed an in silico model of the interaction between the PDZ domain tandem (PDZ1 and PDZ2) of Syntenin-1 and the C-terminal end of SARS-CoV E protein. Our results indicate that the PBM can bind both PDZ domains of Syntenin-1 but shows higher affinity for the PDZ2 domain, and that Syntenin-1 might interact with more than one E protein chain. A similar behavior has been described for the PBM of Syndecan, one of the best-known proteins that interacts with Syntenin-1 [49].

Our model predicts conformational changes after binding, which are allosterically transmitted to other PDZ domains in the Syntenin-1 homodimer. This is coherent with previous reports indicating that Syntenin-1 can adapt its conformation to accommodate different peptides in the PDZ pockets [50] and that both PDZ domains cooperate to facilitate PBM binding [21]. Together with the observed time evolution during molecular dynamics simulations of the pore, this suggests that binding of one PBM to Syntenin-1 might allosterically influence additional binding of a second E protein chain to the opposite PDZ2, and of other downstream signaling proteins to PDZ 1 as previously reported for other PDZ domains [51,52,53].

Following the in silico analysis, we built SARS-CoV mutants in which both individual PBM residues and adjacent residues were mutated to Gly. No difference in virulence was observed in the viruses generated by reverse genetics, compared to the parental virus. Therefore, residues adjacent to the PBM were not the main determinants of SARS-CoV virulence, which suggests that they do not significantly affect the interaction with Syntenin-1. Similarly, none of the individual mutants of the PBM caused attenuation of the virus on their own, suggesting that individual mutations of the PBM are not sufficient to prevent the interaction between the viral PBM and the cellular PDZ domain of Syntenin-1, and therefore the virulence of SARS-CoV. In contrast, the substitution of the four amino acids in the PBM always generated a completely attenuated virus.

As the amino acid sequence of the carboxy-terminus of the E protein from both, SARS-CoV and SARS-CoV-2 is identical (VPDLLV), similar interactions of the carboxy-terminus of the two coronaviruses are expected. Nevertheless, due to the diversification of the sequence in the two following amino acids (EGVPDLLV for SARS-CoV and R-VPDLLV for SARS-CoV-2), it is not possible to fully exclude a different role for the next segment of the E protein, on its interaction with the cellular PDZ domain. In fact, we have demonstrated [54] that a SARS-CoV-2 lacking E protein PBM is fully attenuated in vivo and grows 104-fold lower than the parental virus. In contrast, SARS-CoV lacking E protein PBM, showed no titer reduction. This data reinforces the possibility of different roles of the PBM adjacent amino acids in the SARS-CoV-2 virus.

SARS-CoV PBM binding to PDZ cellular motifs is responsible for virus virulence. In fact, viral PBM may bind to over 400 cellular proteins containing PDZ domains. The role of SARS-CoV 3a and E proteins was studied. E protein PBM was necessary for virulence, whereas the presence or absence of the homologous motif in the 3a protein did not affect virus pathogenicity. Mutagenesis analysis and in silico modeling were used to study the extension of the SARS-CoV E protein PBM and alanine and glycine scanning were performed, revealing that the E protein terminal amino acids (DLLV) were essential for virus virulence and viability. In addition, the last 20 and 22 E protein amino acids (counting from the last one) also influenced virus growth. The binding of E protein with the PDZ2 domain of the Syntenin-1 homodimer induced conformational changes in both PDZ domains 1 and 2 of the dimer that, most likely, affected the pathogenicity of the coronaviruses.

## Figures and Tables

**Table 1:** Oligonucleotides used to engineer SARS-CoV-E mutants.

Virus	Primer	Sequence (5′-3′) ^1^
E-A[27,29]	SARS-E-A[27,29]-RS SARS-E-A[27,29]-VS	CGTTGGTTTTACTAAGGCCACAGCAACAATATTGCAGCA TGCTGCAATATTGTTGCTGTGGCCTTAGTAAAACCAACG
E-A[20,22]	SARS-E-A[20,22]-RS SARS-E-A[20,22]-VS	AACACGCGAGTAGACAGCAACGGCTGGTTTTACTAAACT AGTTTAGTAAAACCAGCCGTTGCTGTCTACTCGCGTGTT
E-A[17,18]	SARS-E-A[17,18]-RS SARS-E-A[17,18]-VS	CTGATTTTTAACACGGGCGGCGACGTAAACCGTTGG CCAACGGTTTACGTCGCCGCCCGTGTTAAAAATCTG
E-A[13,14]	SARS-E-A[13,14]-RS SARS-E-A[13,14]-VS	TTCAGAAGAGTTCAGGGCGGCAACACGCGAGTAGAC GTCTACTCGCGTGTTGCCGCCCTGAACTCTTCTGAA
E-A[10,11]	SARS-E-A[10,11]-RS SARS-E-A[10,11]-VS	AGGAACTCCTTCAGAGGCAGCCAGATTTTTAACACG CGTGTTAAAAATCTGGCTGCCTCTGAAGGAGTTCCT
E-A[8,9]	SARS-E-A[8,9]-RS SARS-E-A[8,9]-VS	AAGATCAGGAACTCCGGCGGCAGAGTTCAGATTTTT AAAAATCTGAACTCTGCCGCCGGAGTTCCTGATCTT
E-G[1]	SARS-E-G[1]-RS SARS-E-G[1]-VS	TAGTTAGTTCGTTTAGCCCAGAAGATCAGGAAC GTTCCTGATCTTCTGGGCTAAACGAACTAACTA
E-G[2]	SARS-E-G[2]-RS SARS-E-G[2]-VS	TTAGTTCGTTTAGACCCCAAGATCAGGAACTCC GGAGTTCCTGATCTTGGGGTCTAAACGAACTAA
E-G[3]	SARS-E-G[3]-RS SARS-E-G[3]-VS	GTTCGTTTAGACCAGACCATCAGGAACTCCTTC GAAGGAGTTCCTGATGGTCTGGTCTAAACGAAC
E-G[4]	SARS-E-G[4]-RS SARS-E-G[4]-VS	CGTTTAGACCAGAAGACCAGGAACTCCTTCAGA TCTGAAGGAGTTCCTGGTCTTCTGGTCTAAACG
E-G[5,6]	SARS-E-G[5,6]-RS SARS-E-G[5,6]-VS	TTAGACCAGAAGATCACCACCTCCTTCAGAAGAGTTC GAACTCTTCTGAAGGAGGTGGTGATCTTCTGGTCTAA
E-G[8,9,10]	SARS-E-G[8,9,10]-RS SARS-E-G[8,9,10]-VS	AAGATCAGGAACTCCTCCACCACCGTTCAGATTTTTAA TTAAAAATCTGAACGGTGGTGGAGGAGTTCCTGATCTT

^1^ Mutated nucleotides are underlined.

**Table 2:** Interaction between the residues of E protein PBM and Syntenin-1. The number of hydrogen bonds and contact atoms in the PBM and the binding score for the whole peptide calculated by Xscore as kcal/mol are indicated for representative conformations of peptides of different lengths. It is important to note that increasing lengths also imply additional restrictions, that the interaction is dynamic, and that similar, slightly different conformations may differ in the number and distribution of interactions depending on the relative orientation of side chains, and thus these values are only orientative.

Length, aa	PBM Hydrogen Bonds					PBM Contacts					Xscore kcal/mol
	Val-1	Leu-2	Leu-3	Asp-4	Total	Val-1	Leu-2	Leu-3	Asp-4	Total	
**6**	3	0	0	0	**3**	44	71	16	34	**166**	**−9.20**
**10**	5	1	1	4	**11**	51	22	18	18	**109**	**−9.38**
**15**	4	0	2	2	**8**	50	14	21	9	**94**	**−9.92**

## Data Availability

Data is contained within the article.
